# Two Odorant-Binding Proteins Mediate the Behavioural Response of Aphids to the Alarm Pheromone (*E*)-ß-farnesene and Structural Analogues

**DOI:** 10.1371/journal.pone.0032759

**Published:** 2012-03-12

**Authors:** Yu Feng Sun, Filomena De Biasio, Hui Li Qiao, Immacolata Iovinella, Shao Xiang Yang, Yun Ling, Lea Riviello, Donatella Battaglia, Patrizia Falabella, Xin Ling Yang, Paolo Pelosi

**Affiliations:** 1 Dipartimento di Biologia delle Piante Agrarie, Pisa, Italy; 2 Department of Applied Chemistry, College of Science, China Agricultural University, Key Laboratory of Pesticide Chemistry and Application, Ministry of Agriculture, Beijing, People's Republic of China; 3 Dipartimento di Biologia, Difesa e Biotecnologie Agro-Forestali, Università della Basilicata, Potenza, Italy; Plant and Food Research, New Zealand

## Abstract

**Background:**

Aphids are agricultural pests of great economical interest. Alternatives to insecticides, using semiochemicals, are of difficult applications. In fact, sex pheromones are of little use as aphids reproduce partenogenetically most of the time. Besides, the alarm pheromone, (*E*)-ß-farnesene for a great number of species, is difficult to synthesize and unstable in the environment. The search for novel semiochemicals to be used in population control can be efficiently approached through the study of the olfactory system at the biochemical level. Recently odorant-binding proteins (OBPs) have been shown to play a central role in olfactory recognition, thus becoming the target of choice for designing new semiochemicals.

**Methodology/Principal Findings:**

To address the question of how the alarm message is recognised at the level of OBPs, we have tested 29 compounds, including (*E*)-ß-farnesene, in binding assays with 6 recombinant proteins and in behaviour experiments. We have found that good repellents bind OBP3 and/or OBP7, while non repellents present different spectra of binding. These results have been verified with two species of aphids, *Acyrthosiphon pisum* and *Myzus persicae*, both using (*E*)-ß-farnesene as the alarm pheromone.

**Conclusions:**

Our results represent further support to the idea (so far convincingly demonstrated only in *Drosophila*) that OBPs are involved in decoding the chemical information of odorants and pheromones, and for the first time provide such evidence in other insect species and using wild-type insects. Moreover, the data offer guidelines and protocols for the discovery of potential alarm pheromones, using ligand-binding assays as a preliminary screening before subjecting selected compounds to behaviour tests.

## Introduction

Aphids represent one of the major pests in agriculture, and their populations cannot be controlled without the extensive use of insecticides. However, their chemical communication systems are exceptionally simple, both with respect to the semiochemicals they use and to the proteins involved in their detection. Although aphids comprise a great variety of species, differing in size, shape, colour and host plants, most of them utilize the same alarm pheromone, (*E*)-ß-farnesene [1]–[4], that is released in the presence of danger and induces other individuals of the same as well as of other species to immediately abandon the place. The sex pheromones of aphids are mixtures, with relative proportions typical of the species, of nepetalactone and its related alcohols nepetalactols [5]–[6]. Both types of pheromones are not suitable for a wide use in agriculture. The synthesis of (*E*)-ß-farnesene is complex and expensive and the compound is relatively volatile. Moreover, its persistence in the environment is also reduced by its ease of oxidation, due to the presence of several double bonds in the molecule. It has been shown that in normal conditions of temperature and sunlight about 77% is degraded after 24 hours and only traces are left after 48 hours [7]. It is interesting that a number of plants succeed in keeping away aphids by synthesising (*E*)-ß-farnesene, thus indicating that in principle this compound can be efficiently utilised as aphid repellent [8]. This fact has suggested the idea of producing transgenic *Arabidopsis thaliana* that is able to synthesise (*E*)-ß-farnesene and defend itself from aphids [9], thus paving the way for extending such approach to crop plants. Sex pheromones, on the other hand, are of little use, not only in population control, but also for monitoring purposes, because aphids reproduce most of the time parthenogenetically.

Therefore, we have decided to study the aphid chemoreception system at the biochemical level in order to find new strategies for population control. In particular we have focused our research on odorant-binding proteins (OBPs), soluble proteins secreted in the lymph of chemosensilla [10]–[13]. In recent years, these polypeptides, first regarded as passive carriers for pheromones and odorants, have been recognised as very important elements in the process of chemodetection and recognition. Early experments performed with recombinant PBPs of *Antheraea polyphemus* and *Bombyx mori* suggested that different pheromone components induced specific conformational changes in different PBPs [14] and that electrophysiological response of sensilla to pheromone components depended on the presence of the correct PBP [15]–[16]. More recently, evidence has been provided that the presence of OBPs confers higher sensitivity and specificity to the olfactory system of *B. mori* [17]–[18], while LUSH, one of the OBPs of *Drosophila melanogaster* has been shown to be strictly required for perception of the male pheromone vaccenyl acetate [19]. In the same protein, binding of the pheromone induces a conformational change that triggers specific interaction with the corresponding odorant receptor [20]. This has been very elegantly demonstrated by showing that a mutant of LUSH, mimicking the conformation of the protein when bound to vacceny acetate, efficiently activates the olfactory receptor, even in the absence of the pheromone. At the behavioural level, exchange of two genes encoding OBPs in two species of *Drosophila* produces, to some extent, switching of behaviour towards some fatty acids [21]. Finally, a very recent work analysed the response to several odorant of 17 strains of *Drosophila*, each deficient in one specific OBP [22]. The results clearly show that in each strain the olfactory response was modified in different specific way, in some cases finely tuned to one or two odorants. These fundamental papers not only provide strong evidence that OBPs are involved in odour discrimination, but also indicate that a combinatorial code is utilised by insects to recognise olfactory stimuli. Moreover, they shift the focus from olfactory receptors to OBPs as the proteins responsible for decoding the chemical information carried by odorants and pheromones, and the best target for interfering with the insect's olfactory system.

Further support to the key role of OBPs in odour discrimination has been provided in two species of mosquitoes, *Anopheles gambiae* and *Culex quinquefasciatus*, where silencing genes for OBPs selectively abolishes response to indole [23]–[24]. We can therefore confidently attempt to relate the binding specificities of OBPs in a given species with behavioural responses.

The genome sequencing of the pea aphid *Acyrthosiphon pisum* has revealed the presence of 15 genes encoding OBPs [25]. In a first investigation limited to three OBPs, we have found that (*E*)-ß-farnesene and related compounds bind specifically to only OBP3, suggesting not only that this protein might be involved in the recognition of the alarm pheromone, but also that activation of other OBPs could produce a response pattern that is no longer recognised as an alarm signal [26].

We have also synthesised new insecticides combining classical aphicidal moieties with a terpene region mimicking (*E*)-ß-farnesene. Such compounds retain insecticidal activity, as well as specific binding to OBP3, suggesting that they might be also endowed with repellent properties [27].

Here we describe the bacterial expression and characterization of three additional OBPs of aphids. We have also measured the binding properties of 29 pure chemicals with six recombinant OBPs and their repellent activity in two species of aphids. On the basis of the results obtained, we suggest that OBP3 and OBP7 are the proteins responsible for mediating the perception of the alarm pheromone (*E*)-ß-farnesene and of other repellents, and provide guidelines for the discovery and design of new aphid repellents.

## Materials and Methods

*Insects - Acyrthosiphon pisum* (Harris) was reared on potted broad bean plants (*Vicia faba* L) in a climatic chamber at 20±1°C, 75±5% RH, under an LD 18∶6 h photoperiod. Aphids culture was started in 1985, with a few hundred specimens originally collected from lucerne (*Medicago sativa* L) fields, in Southern Italy (Eboli, SA). Wingless adults were used in behaviour experiments.

*Myzus persicae* (Sulzer) culture was reared under constant climatic conditions (20±1°C, 60–70% RH, natural lighting) on wild cabbage (*Brassica oleracea* L) in the greenhouse of the Institute of Plant Protection, Chinese Academy of Agricultural Sciences, Beijing. The fourth-instar and wingless adults *M. persicae* were used in behavioural test.

*Reagents and ligands* - All enzymes were from New England Biolabs. Oligonucleotides were custom synthesized at Eurofins MWG GmbH, Ebersberg, Germany. All other chemicals, unless otherwise specified, were purchased from Sigma-Aldrich and were of reagent grade. All CAU ligands were synthesised using our previously described methods [27]. The synthetic details will be reported in another publication. The other ligands, with the exception of geranyl acetate and farnesol (Sigma-Aldrich), as well as (*E*)-ß-farnesene (Bedoukian Research, Danbury, CT, USA), were prepared using well established conventional routes.

*RNA extraction and cDNA synthesis* - Total RNA was extracted from TRI® Reagent (Sigma-Aldrich), following the manufacter's protocol. cDNA was prepared from total RNA by reverse transcription, using 200 units of SuperScript™ III Reverse Transcriptase (Invitrogen) and 0.5 µg of an oligo-dT primer in a 50 µL total volume. The mixture also contained 0.5 mM of each dNTP (GE-Healthcare), 75 mM KCl, 3 mM MgCl_2_, 10 mM DTT and 0.1 mg/mL BSA in 50 mM Tris-HCl, pH 8.3. The reaction mixture was incubated at 50°C for 60 min and the product was directly used for PCR amplification or stored at −20°C.

*Polymerase chain reaction* - Aliquots of 1 µL of crude cDNA were amplified in a Bio-Rad Gene CyclerTM thermocycler, using 2.5 units of *Thermus aquaticus* DNA polymerase (GE-Healthcare), 1 mM of each dNTP (GE-Healthcare), 1 µM of each PCR primer, 50 mM KCl, 2.5 mM MgCl_2_ and 0.1 mg/mL BSA in 10 mM Tris-HCl, pH 8.3, containing 0.1% v/v Triton X-100. At the 5′ end, we used specific primers corresponding to the sequence encoding the first six amino acids of the mature protein. Signal peptides were predicted using the on-line programme Signa-P and not included in the segment to be amplified. The primers also contained an *Nde* I restriction site, for ligation into the expression vector and providing at the same time the ATG codon for an additional methionine in position 1. At the 3′ end specific primers were used, encoding the last six amino acids, followed by a stop codon and an *Eco*R I restriction site for ligation into the expression vector. Therefore, we used the following primers for the each protein (enzyme restriction sites are underlined):

fw *Apis* OBP6: 5′-AACATATGATGCCAAATATATTACC-3′

rv *Apis* OBP6: 5′-GTCCATGGTTAGATTAATTTAGGTGGTGA-3′

fw *Apis* OBP7: 5′-AACATATGTACTTGAGTGAAGCGGC-3′

rv *Apis* OBP7: 5′-GTGAATTCCTAGAGTGGTAGAAACTC-3′

fw *Apis* OBP10: 5′-AACATATGACACGACCACAACCAGA-3′

rv *Apis* OBP10: 5′-GTGAATTCTCACAGTGGTAGGAGTGC-3′

After a first denaturation step at 95°C for 5 min., we performed 35 amplification cycles (1 min. at 95°C, 30 sec. at 50°C, 1 min. at 72°C) followed by a final step of 7 min at 72°C. In all experiments we obtained amplification products of 350–500 bp, in agreement with the expected sizes.

*Cloning and sequencing* - The crude PCR products were ligated into a pGEM (Promega) vector without further purification, using a 1∶5 (plasmid∶insert) molar ratio and incubating the mixture overnight, at room temperature. After transformation of *E. coli* XL-1 Blue competent cells with the ligation products, positive colonies were selected by PCR using the plasmid's primers SP6 and T7 and grown in LB/ampicillin medium. DNA was extracted using the kit GFX Micro Plasmid Prep (GE-Healthcare) and custom sequenced at Eurofins MWG (Martinsried, Germany).

*Cloning in expression vectors* - pGEM plasmids containing the appropriate sequences were digested with *Nde* I and *Eco*R I restriction enzymes for two hours at 37°C and the digestion products were separated on agarose gel. The obtained fragments were purified from gel and ligated into the expression vector pET30b (Novagen, Darmstadt, Germany), previously linearized with the same enzymes. The resulting plasmids were sequenced and shown to encode the mature proteins.

*Preparation of the proteins* - For expression of recombinant proteins, each pET-30b vector containing the appropriate OBP sequence was used to transform BL21(DE3)pLysS *E. coli* cells. Protein expression was induced by addition of IPTG to a final concentration of 0.4 mM when the culture had reached a value of O.D._600_ = 0.8. Cells were grown for further 2 hours at 37°C, then harvested by centrifugation and sonicated. After centrifugation, OBPs were present as inclusion bodies. To solubilise them, the pellet from 1 L of culture was dissolved in 10 mL of 8 M urea, 1 mM DTT in 50 mM Tris buffer, pH 7.4, then diluted to 100 mL with Tris buffer and dialysed three times against Tris buffer.

Purification of the proteins was accomplished by combinations of chromatographic steps on anion-exchange resins, such as DE-52 (Whatman) and QFF (GE-Healthcare), followed by gel filtration on Sephacryl-100 or Superose-12 (GE-Healthcare) along with standard protocols previously adopted for other odorant-binding proteins [28]–[29]

*Fluorescence measurements* - Emission fluorescence spectra were recorded on a Jasco FP-750 instrument at 25°C in a right angle configuration, with a 1 cm light path quartz cuvette and 5 nm slits for both excitation and emission. The protein was dissolved in 50 mM Tris-HCl buffer, pH 7.4, while ligands were added as 1 mM methanol solutions.

*Fluorescence binding assays* - To measure the affinity of the fluorescent ligand N-phenyl-1-naphthylamine (1-NPN) to each protein, a 2 µM solution of the protein in 50 mM Tris-HCl, pH 7.4, was titrated with aliquots of 1 mM ligand in methanol to final concentrations of 2–16 µM. The probe was excited at 337 nm and emission spectra were recorded between 380 and 450 nm. The affinity of other ligands was measured in competitive binding assays, using 1-NPN as the fluorescent reporter at 2 µM concentration and 2–16 µM concentrations of each competitor.

For determining binding constants, the intensity values corresponding to the maximum of fluorescence emission were plotted against free ligand concentrations. The curves were drawn using the Prism software, that allowed calculation of the dissociation constants and the stoichiometry of binding, assuming that the protein was 100% active. Dissociation constants of the competitors were calculated from the corresponding IC_50_ values, using the equation: K_D_ = [IC_50_]/1+[1-NPN]/K_1-NPN_, [1-NPN] being the free concentration of 1-NPN and K_1-NPN_ being the dissociation constant of the complex Protein/1-NPN.

*Behaviour experiments* - Behavioural response of *A. pisum* was investigated with a glass Y-tube olfactometer (2.7 cm uniform diameter, 26.5 cm main body length, and 16.5 cm branch length). In the centre of the Y tube, a Y-shaped copper wire was positioned to facilitate the movement of the aphids towards one or the other ends of the olfactometer [30].

An airflow (0.5 L/min) was introduced into each arm of the olfactometer through glass stimulus chamber (an odour source adapter), attached to each of the two ending arms. In this way, two well-separated laminar air flows were generated in the olfactometer.

In each test 1 µL of hexane solution of each chemical, concentration 0.5%, was placed in the glass stimulus chamber of the “treatment” arm. As a control, 1 µL of hexane was placed in the glass stimulus chamber of the “control” arm of the olfactometer. Experiments were performed at room temperature (20–25°C). The olfactometer was washed with detergent and water before each experiment.

Groups of twenty apterous adult aphids were introduced at the bottom of the Y-shaped copper wire and let free to walk to either arm at the Y-junction.

After 15 minutes, the number of aphids in the treatment and control sides of the olfactometer were counted. Aphids that did not move and remained at the base of the Y tube were recorded but their number was not considered in the analysis. We performed 4 replications with each compound.

Behaviour experiments with *M. persicae* (Sulzer) were performed in the same conditions described for *A. pisum*, except for the following differences. The Y-tube olfactometer was 1.0 cm in diameter, and both the main body and the two branches were 5.0 cm long. The air flow of humidified air was 0.2 L/min. The aphid fourth instars and wingless adults were used in the bioassay.

*Statistical analysis* - The repellency of each compound was estimated by the repellency index (R), a modification of the excess proportion index [31]. The repellency index is calculated by the formula R = (C−T)/(C+T), where T indicates the number of aphids in the arm with the compound to be tested and C those in the control arm. The repellency of any single compound tested was then analysed on the basis of total number of aphids in each olfactometer's arm by the chi-square goodness-of-fit test [32]. This test assesses whether a significant difference exists between the observed number of aphids in each arm of the olfactometer and the expected number based upon the null hypothesis of a 50∶50 aphid's distribution. Moreover, the behavioural responses to the 29 compounds tested were compared between them by the chi-square test (df = 28), separately for *M. persicae* and *A. pisum* [32]. As differences among the behavioural responses of the two aphid species to the chemicals tested were significant, to answer the question of where the differences could be found, each contingency table was partitioned into subtables and each of them was analysed. First we compared the 17 chemicals binding OBP3 and/or OBP7, among them (d.f. = 16), and the other 12 chemicals (binding more OBPs or none) among them (d.f. = 11). Then we grouped the data according to the OBPs bound, and we repeated the chi-square test comparing groups of chemicals. The chi-square test was performed with SigmaStat 3.1 software.

*Molecular modelling* – Three-dimensional models of OBPs were generated using the on-line programme SWISS MODEL [33]–[35]. For OBP3 we used the structure of the PBP of *Leucophaea maderae* (PDB: 1ORG_A [36] as a template. Amino acid identity between the two proteins is 23%. For OBP7 the template was the structure of *Drosophila* Lush acc. No. 3b6xB [37] (identity between the two proteins: 17%). Models were displayed using the SwissPdb Viewer programme “Deep-View” [34] (http://www.expasy.org/spdbv/).

## Results and Discussion

### Selection of the OBPs to be expressed

Our research was aimed at understanding the molecular mechanisms responsible for the detection of (*E*)-ß-farnesene in aphids with specific attention to the roles of OBPs. Following our previous characterization of three OBPs in the pea aphid *A. pisum* [26], we decided to express three additional proteins of the same family and measure their binding properties. The recent sequencing of the genome of the pea aphid *A. pisum* allowed the identification and annotation of all the genes encoding OBPs. In total 15 genes encoding such proteins are reported, but for two of them (OBP14 and OBP15) the partial sequences published are too short to allow their assignment to the OBP family [25]. Of the remaining 13 sequences, three are closely related (OBP3, OBP11 and OBP12), while the others are extremely divergent with percent of identical amino acids around 10% in most cases. Moreover, OBP2, OBP4, OBP5 and OBP6 are longer than other OBPs and contain extra cysteines besides the six characteristic of all insect OBPs. Figure S1 reports the amino acid sequences alignment for the13 OBPs of the pea aphid *A. pisum*.

Based on such analysis, we decided to express OBPs 6, 7 and 10, that, together with OBPs 1, 3 and 8, previously described [26] could be representative of the OBP repertoire in aphids. Particularly interesting is OBP7 for being similar to OBP3 in its binding pocket, although the two proteins only share 15% of their amino acids when comparing full mature sequences.

**Figure 1:**
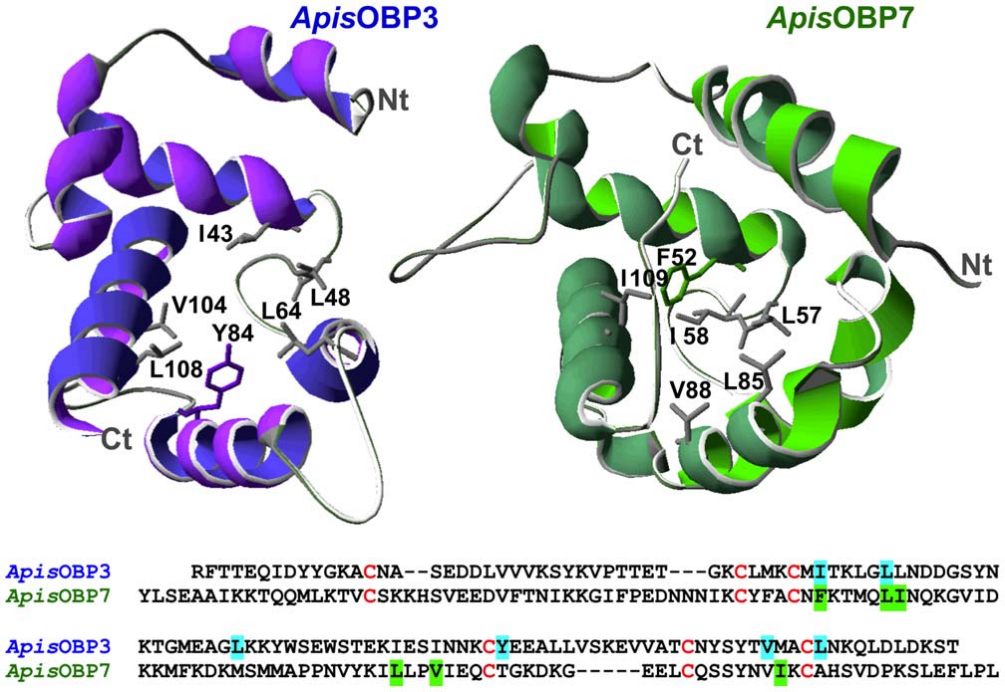
Sequences and three-dimensional models of *A. pisum* OBP3 and OBP7. The two OBPs, despite a low sequence identity of 15%, show a remarkable similarity in their overall folding with special reference to their binding pockets. In both cases, the hydrophobic cavity is lined with several branched chain amino acids (Val, Ile, Leu) and an aromatic residue, Tyr84 in OBP3 and Phe52 in OBP7 (highligthed in the sequences). Three-dimensional models of OBPs were generated using the on-line programme SWISS MODEL [33]–[35]. For OBP3 we used the structure of the PBP of *Leucophaea maderae* (PDB: 1ORG_A [36] as a template. Amino acid identity between the two proteins is 23%. For OBP7 the template was the structure of *Drosophila* LUSH (acc. No. 3b6xB [37], identity between the two proteins: 17%). Models were displayed using the SwissPdb Viewer programme “Deep-View” [34] (http://www.expasy.org/spdbv/). Residues shown in the binding pockets are highlighted in the sequences.

In our previous paper [26] we reported that (*E*)-ß-farnesene and some structural analogues (farnesol and 3,7-dimethyloctyl acetate) exhibit specific binding to OBP3 and suggested that this protein could be involved in the perception of the alarm pheromone. We also hypothesised that the branched amino acids (valine, leucine and isoleucine) lining the binding pocket of OBP3 could establish close interactions with the terpene branched chain of (*E*)-ß-farnesene, while the aromatic ring of Tyr84 would match the two terminal conjugated double bonds of the same ligand. As shown in Figure 1, OBP7 (unlike the other OBPs of *A. pisum*) also presents a set of branched residues in its binding pocket and an aromatic residue, Phe52. Although the two aromatic amino acids are situated on opposite sites in the binding pockets of the two proteins, their positions relative to the other residues lining the binding cavity suggest that OBP3 and OBP7 could exhibit some similarity in their ligand-binding specificities.

Therefore, to test our hypothesis that (*E*)-ß-farnesene and structurally related compounds could preferentially bind OBP3 and OBP7 and that these two proteins might be responsible for detecting the alarm pheromone, we have expressed OBP7, OBP6 and OBP10, and analysed the binding specificities of the six proteins so far available.

### Expression and purification of the OBPs

**Figure 2:**
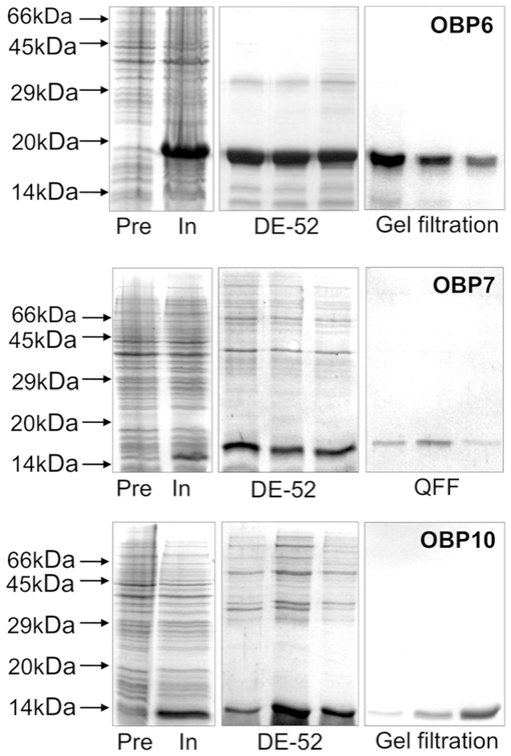
Expression and purification of *A. pisum* OBP6, OBP7 and OBP10. Proteins were expressed in BL21(DE3)pLysS *E. coli* cells transformed with pET-30b vector containing the appropriate OBP sequence. Recombinant proteins were obtained in high yields (about 20 mg/L) as inclusion bodies and were solubilised by denaturation and refolding as described in the Materials and Methods sections. Purification was accomplished by combinations of chromatographic steps on anion-exchange resins, such as DE-52 (Whatman) and QFF (GE-Healthcare), followed by gel filtration on Sephacryl-100 or Superose-12 (GE-Healthcare) along with standard protocols previously adopted for other odorant-binding proteins.

Figure 2 summarizes the bacterial expression and purification of OBPs 6, 7 and 10. As in the case of other insect OBPs, these proteins were produced in high yields (at least 20 mg/L of culture) and easily purified by conventional chromatographic methods. As the proteins were produced as inclusion bodies, their solubilisation was accomplished by denaturation and refolding, according to protocols that have been verified to afford the proteins in their active forms [28], [37].

### Ligand-binding studies

To investigate the binding specificity of each OBP, we used two sets of potential ligands. The first series comprises a structurally homogeneous group of synthetic compounds, each containing an aromatic moiety, endowed with insecticidal activity, linked to a geranyl group, that mimicks the structure of (*E*)-ß-farnesene. The synthetic routes for compounds of this type have been previously described [26]. The second series includes eight pure chemical compounds, the alarm pheromone (*E*)-ß-farnesene and some structurally-related substances, such as farnesol, 3,7-dimethyloctyl acetate, 3,7-dimethyloctyl benzoate and geranyl acetate, as well as other compounds of different structure, namely n-butyl benzoate, n-hexyl benzoate and isopentyl 4-phenylbutyrate.

**Figure 3:**
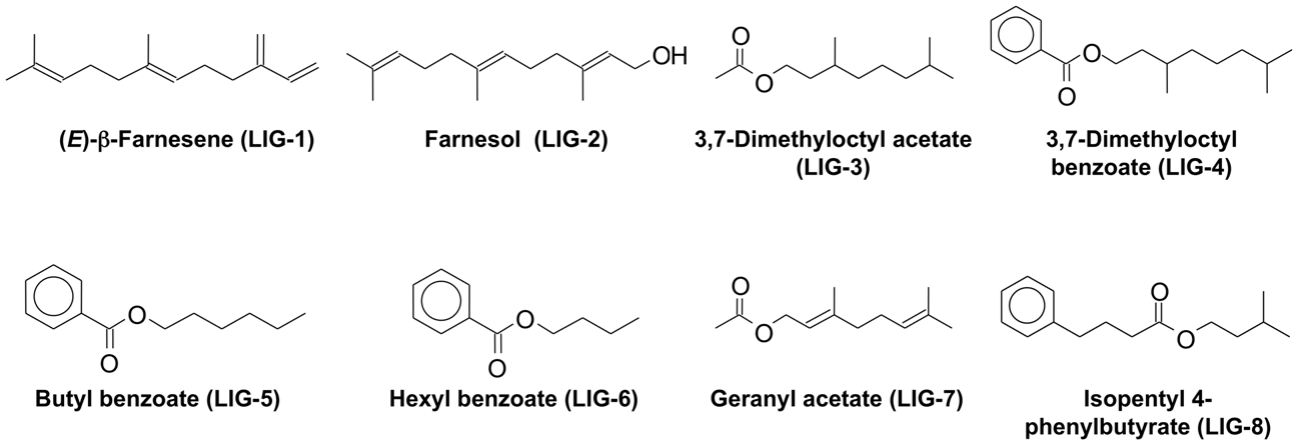
Chemical structures of the general odorants used in binding experiments.

**Figure 4:**
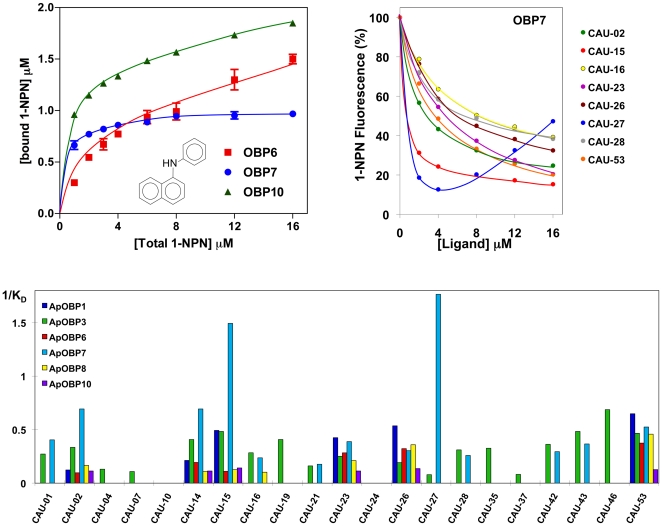
Binding of CAU ligands to the six *A. pisum* OBPs. *Upper left panel*. Binding of 1-NPN to the recombinant OBP6, OBP7 and OBP10. 2 µM solutions of each protein in 50 mM Tris-HCl, pH 7.4 was titrated with 1 mM solution of 1-NPN in methanol to final concentrations of 2–16 µM. The data, averages of three replicates, were analysed using Prism software and indicate in each protein the presence of a single binding site. Dissociation constants were 6.3 (SEM 1.0), 0.54 (SEM 0.05) and 1.2 (SEM 0.15) for OBPs 6, 7 and 10, respectively. The same software showed that saturation occurs at one binding site/monomer for OBP6 and OBP7, but at one binding site/dimer in the case of OBP7. *Upper right panel*. Competition binding assays of selected CAU ligands to OBP7. In each experiment a mixture of the protein and 1-NPN in Tris, both at the concentration of 2 µM, was titrated with 1 mM methanol solutions of the competing ligands to final concentrations of 2–16 µM. Fluorescence intensities are reported as percent of the values in the absence of competitor. *Lower panel*. Reverse values of the dissociation constants measured with all 21 CAU ligands and the six *A. pisum* OBPs. The values of the dissociation constants are reported as supplementary information in Table S1. The structures of the 21 CAU ligands are reported in Table S3.

**Figure 5:**
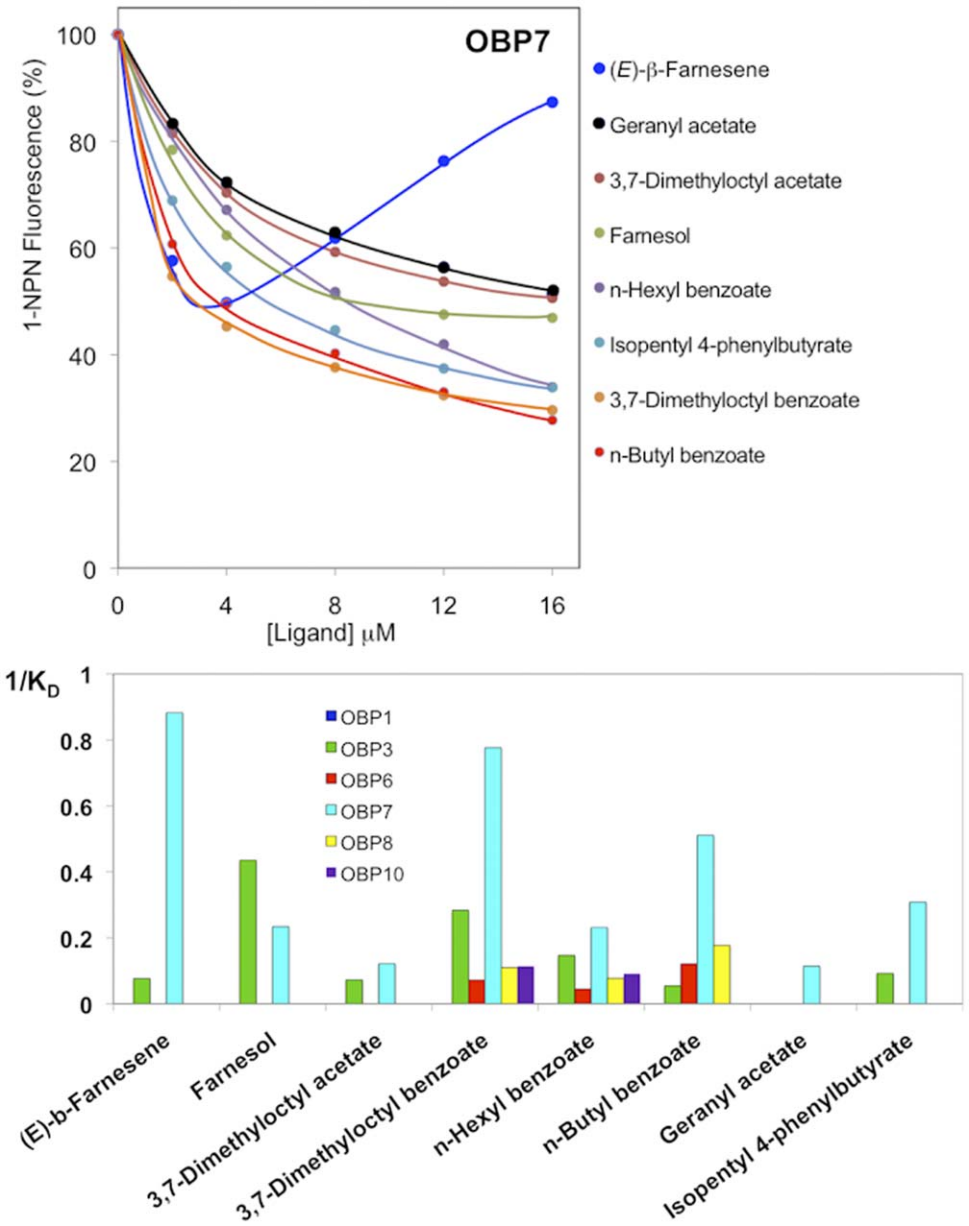
Binding of general odorants to the six *A. pisum* OBPs. *Upper panel*. Competition binding curves of 8 ligands to OBP7. *Lower panel*. Reverse values of the dissociation constants measured with the same ligands and the six *A. pisum* OBPs. The values of the dissociation constants are reported as supplementary information in Table S1. The structures of the 8 ligands are reported in Figure 3.

The structures of all the ligands used are reported in Table S3 and Figure 3. The results of binding experiments are reported in Figure 4 and 5, while Table S1 lists the values of [IC]_50_ measured with all the ligands and the six OBPs, together with the calculated dissociation constants.

All three newly expressed proteins (OBP6, OBP7 and OBP10) bind the fluorescent probe with good affinity (Figure 4, upper left panel), as most of insect OBPs, thus allowing other ligands to be tested in competitive binding assays. The upper right panel of Figure 4 reports, as examples, the displacement curves of 1-NPN from the complex with OBP7 by increasing amounts of selected ligands from the first series (CAU). With some ligands, as CAU 27, we observed an increase of 1-NPN fluorescence at higher concentrations of the ligand. A likely explanation of this phenomenon would suggest that above a certain concentration some ligands might form micelles entrapping molecules of 1-NPN, that, being in a hydrophobic environment, would produce a fluorescence peak in the same region of the spectrum as that relative to 1-NPN bound to the protein. This hypothesis is supported by the fact that such peak is observed when analysing a mixture of 1-NPN and ligand in the absence of the protein. In the lower panel the reverse values of the dissociation constants of all the chemicals belonging to the first series, measured with the six proteins are plotted and compared. Figure 5 reports parallel data obtained with the same proteins and eight general odorants (second series).

Each OBP, as expected, displayed a different spectrum of binding with a broad specificity towards a number of ligands. Looking at the selectivity of each ligand towards the six OBPs, we can observe that some compounds, including (*E*)-ß-farnesene and structurally similar molecules, bind only OBP3 and/or OBP7, while others also exhibit measurable affinity to the other OBPs or else do not bind any of the proteins tested.

### Behaviour experiments

To investigate whether OBP3 or OBP7 or both could be involved in detecting the signal of alarm pheromones, we performed behaviour experiments with all the compounds used in binding studies, using two species of aphids, *A. pisum* and *M. persicae*. Both species use (*E*)-ß-farnesene as the alarm pheromone [2], [38]. Moreover, OBPs are exceptionally well conserved in many aphid species [25]. In particular the amino acid sequence of OBP3 of *M. persicae* presents only three substitutions with respect to that of *A. pisum*, all occurring outside the binding pockets. The sequences of OBP7 are more divergent in the two species (90% identity), but only two substitutions (L57M and V88A) are located inside the binding site and are not likely to modify appreciably the hydrophobic microenvironment.

For our experiments we used a Y tube olfactometer, as described in the Materials and Methods section. Aphids of the fourth instar as well as adults were used and the chemicals were tested at the concentration of 0.5%.

In all experiments we observed that a certain number of aphids did not make any choice between the two arms of the olfactometer (chemical vs solvent), but remained at the base of the tube, even in the control experiments, where the solvent was used at both arms of the tube. This phenomenon, that has been previously reported [39], [40], could be due to several reasons, from inability of those individuals to smell the compound to a particularly high sensitivity that enabled them to detect the chemical already at the base of the tube, or to the reduced motility caused by fungal infections [41], [42].

**Figure 6:**
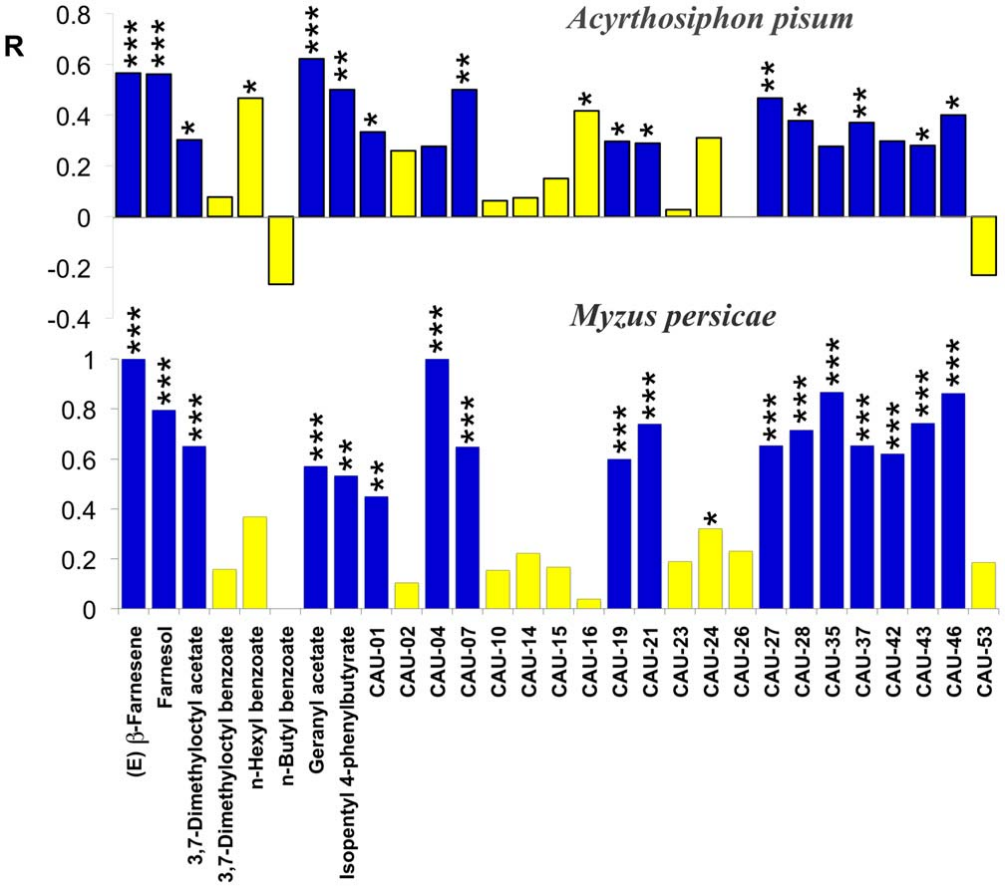
Repellency of the ligands used in binding assays against *A. pisum* and *M. persicae*. Compounds that exhibited affinity to either OBP3 or OBP7 or both proteins are in blue, the others are in yellow. The repellency index R was calculated by the formula R = (C−T)/(C+T), where T indicates the number of aphids in the arm with the compound to be tested and C those in the control arm; asterisks indicate that the repellence observed is statistically significant (chi-square goodness-of-fit test): * P<0.05, ** P<0.01, ***P<0.001. *A. pisum*: (*E*)-β-Farnesene χ^2^ = 14.70; Farnesol χ^2^ = 13.25; 3,7-Dimethyloctyl acetate χ^2^ = 4.05; 3,7-Dimethylocty benzoate χ^2^ = 0.17; n-Hexyl benzoate χ^2^ = 6.53; n-Butyl benzoate χ^2^ = 2.13; Geranyl acetate χ^2^ = 14.72; Isopentyl 4-phenylbutyrate χ^2^ = 10.00; CAU-01 χ^2^ = 4.24; CAU-02 χ^2^ = 1.92; Cau-04 χ^2^ = 3.70; CAU-07 χ^2^ = 8.00; CAU-10 χ^2^ = 0.12; CAU-14 χ^2^ = 0.30; CAU-15 χ^2^ = 0.9; CAU-16 χ^2^ = 4.17; CAU-19 χ^2^ = 4.74; CAU-21 χ^2^ = 3.86; CAU-23 χ^2^ = 0.06; CAU-24 χ^2^ = 2.92; CAU-26 χ^2^ = 0.00; CAU-27 χ^2^ = 10.04; CAU-28 χ^2^ = 6.59; CAU-35 χ^2^ = 3.70; CAU-37 χ^2^ = 7.41; CAU-42 χ^2^ = 3.89; CAU-43 χ^2^ = 3.92; CAU-46 χ^2^ = 6.4; CAU-53 χ^2^ = 0.83. *M. persicae*: (*E*)-β-Farnesene χ^2^ = 50.04; Farnesol χ^2^ = 25.32; 3,7-Dimethyloctyl acetate χ^2^ = 16.90; 3,7-Dimethylocty benzoate χ^2^ = 0.56; n-Hexyl benzoate χ^2^ = 2.78; n-Butyl benzoate χ^2^ = 0.12; Geranyl acetate χ^2^ = 13.71; Isopentyl 4-phenylbutyrate χ^2^ = 8.53; CAU-01 χ^2^ = 8.10; CAU-02 χ^2^ = 0.36; Cau-04 χ^2^ = 36.06; CAU-07 χ^2^ = 21.80; CAU-10 χ^2^ = 1.23; CAU-14 χ^2^ = 1.78; CAU-15 χ^2^ = 1.00; CAU-16 χ^2^ = 0.08; CAU-19 χ^2^ = 16.59; CAU-21 χ^2^ = 25.10; CAU-23 χ^2^ = 1.39; CAU-24 χ^2^ = 5.58; CAU-26 χ^2^ = 2.16; CAU-27 χ^2^ = 19.56; CAU-28 χ^2^ = 21.43; CAU-35 χ^2^ = 22.53; CAU-37 χ^2^ = 19.56; CAU-42 χ^2^ = 16.10; CAU-43 χ^2^ = 17.67; CAU-46 χ^2^ = 22.35; CAU-53 χ^2^ = 1.00.

For our calculations we decided to ignore such individuals and included only those that had reached, at the end of the experiment, one of the two arms. The repellency of each compound (R) is calculated by the formula R = (C−T)/(C+T), where T indicates the number of aphids in the arm with the compound to be tested and C those in the control arm. Therefore, a value of R = 1 indicates that all the insects that made a choice were found in the control arm, while R = 0 corresponds to a situation with the aphids distributed equally between the two arms and indicates no effect of the tested substance. The results of the behaviour tests with the two species of aphids are reported in Figure 6. Compounds specifically binding OBP3 and/or OBP7 are indicated with blue colour bars, the others with yellow bars. We can observe that the behavioural responses of the two aphid species follow similar patterns, although *M. persicae* showed a stronger response to repellents than *A. pisum*. All compounds binding OBP3 and/or OBP7 (blue colour in the graphic) were significant repellents for *M. persicae* (Figure 6). For *A. pisum*, the same compounds were also repellents except CAU-04, CAU-35 and CAU-42. However, in these cases the calculated χ^2^ (CAU-04 χ^2^ = 3.70; CAU-35 χ^2^ = 3.70; CAU-42 χ^2^ = 3.39) were very close to the value corresponding to α = 0.05 level of significance (χ^2^ = 3.84). All compounds binding additional OBPs (yellow colour in the graphic) were not repellents, except CAU-24 in the case of *M. persicae*, and CAU-16 and n-hexyl benzoate in the case of *A. pisum*. A comparison among behavioural responses elicited by all the compounds tested is reported in Table S2.

**Figure 7:**
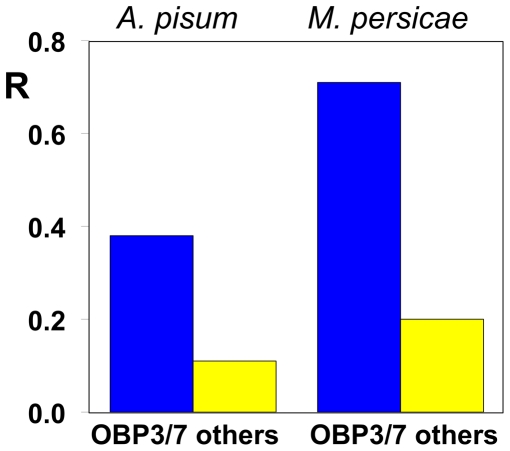
Comparison between the repellency of the two groups of chemicals. In both aphid species, chemicals binding exclusively OBP3 and/or OBP7 are much stronger repellents than other ligands with different spectra of binding. For *A. pisum*: χ^2^ = 13.970, d.f. 1, P<0.001; for *M. persicae*: χ^2^ = 96.044, d.f. 1, P<0.001.

As shown in Figure 7 and Table S2, the repellency elicited by ligands of additional OBPs considered as a whole was much lower than the repellency elicited by (*E*)-ß-farnesene and ligands of OBP3 and/or OBP7 as a whole, for both aphid species (*A. pisum* χ^2^ = 13.970, d.f. 1, P<0.001; *M. persicae* χ^2^ = 96.044, d.f. 1, P<0.001) (Figure 7).

In summary, we can say that compounds selectively binding OBP3 and/or OBP7 have a much higher probability of being repellents than compounds binding other OBPs or none. This suggests that OBP3 and OBP7 could mediate the perception and the consequent behavioural response to (*E*)-ß-farnesene and other repellents, and that only a selective activation of one or both of these OBPs is interpreted as a specific signal of danger. Moreover, such results allow us to use this model as a working hypothesis to further investigate the mechanism of alarm pheromone recognition by aphids and devise strategies and protocols to discover new aphid repellents.

### Conclusions

In our previous paper we had suggested that OBP3 could be involved in the perception of (*E*)-ß-farnesene. The novel concept that we had proposed was that recognition of the alarm pheromone could be based not on a particularly high affinity of this compound for OBP3, but rather on the specific binding to only such OBP among the three tested in that report [26]. In this work we support this model with behavioural experimental evidence and include a second protein (OBP7) that could mediate perception of the alarm pheromone. OBP7 is very similar to OBP3, but only limited to their binding pockets, while identity between the two proteins is not more than 11%. Most important, however, the behaviour data reported for two species of aphids, indicate a consistency between repellency and binding characteristics. Based on these six OBPs, that are representative of the small aphids repertoire of these polypeptides, we can propose a model that allows us to predict, with reasonable confidence, whether a new chemical compound could be a repellent (for aphids that use (*E*)-ß-farnesene as the alarm pheromone), based on binding experiments. In particular, we have shown that repellency correlates well with exclusive affinity to OBP3 or OBP7 or both. A measurable affinity to other OBPs could significantly modify the olfactory map produced in the antennal lobes to impair recognition of the stimulus as similar to (*E*)-ß-farnesene.

The idea that each olfactory stimulus generates a complex map at the level of antennal lobes and that the map is recognised by the brain, rather than its components, is certainly not new, both in insects and in vertebrates. Each glomerulus receives the signals from each type of olfactory receptors and the map in the antennal lobe is basically a processed version of the map generated at the periferal level by olfactory receptors. Now, in the light of the new specific role of OBPs in olfactory perception [14]–[22] and based on the results here presented, we can identify a level, between odorants and olfactory receptors, where decodification of the chemical message is performed by OBPs.

However, our model is a crude simplification of the more complex biological system. The correlation between binding data and behaviour, that we have presented in this work, although statistically significant, is not perfect. The occurrence of few exceptions to the rule we propose can be related to several factors. First, other OBPs, among those we have not examined, or also CSPs or other still unknown binding proteins, could be involved in building the olfactory image of (*E*)-ß-farnesene and other repellents. Second, our criterion of considering as ligands only those compounds that could displace 50% of the bound 1-NPN at a concentration lower than 20 µM could be rather arbitrary and affect to some extent the classification of a compound as “ligand”. Finally, we should be aware that binding activity, as measured “in vitro” does not faithfully reproduce the “in vivo” conditions, particularly in terms of protein concentration. Given the above limitations and caveats, however, our data provide for the first time a good correlation between behavioural effects and binding properties of odorants and further support the recent view that OBPs in insects are responsible for recognising the different semiochemicals. Moreover, they can provide indications and guidelines for designing and screening new repellents for aphids. In fact, potential repellents could be first tested in simple and rapid binding experiments with the six OBPs, and then only those binding OBP3 and/or OBP7 could be further screened in more expensive and time-consumeing behaviour experiments. Similar approaches could also be devised to tackle analogous problems in other insect species.

## Supporting Information

Figure S1 **Alignment of amino acid sequences of the OBPs of** ***A. pisum*** **.** The 13 sequences predicted by the genome are very divergent and, in order to align the six conserved cysteines, several gaps had to be introduced. OBP2 is much longer and, together with OBP4, OBP5 and OBP6, present several additional cysteines. Signal peptides (where prediction was feasible) are underlined.
(TIFF)

Table S1 **Binding properties of the ligands utilised in ligand binding assays and behaviour experiments.** For each protein, [IC]_50_ reports the concentration of ligand that halves the initial fluorescence intensity (both protein and 1-NPN were used at the concentration of 2 µM). Dissociation constants are calculated as reported in the Materials and Methods section. The structures of “CAU” ligands are reported in Table 1, those of “LIG” chemicals in Figure 3. Blank cells indicate values of [IC]_50_ higher than 30 µM. For these compounds dissociation constants have not been calculated.
(DOC)

Table S2 **Comparison among behavioural responses elicited by all compounds tested separately or as a function of OBPs bound.** Asterisks indicate that the difference is statistically significant: * P<0.05, ** P<0.01, ***P<0.001.
(DOC)

Table S3 **Structures of the “CAU” ligand utilised in binding assays and behaviour experiments.** Abbreviations are: Me: methyl; Pr: propyl; i-Pr: isopropyl; t-Bu: *tert*-butyl; Phe: phenyl; p-NO_2_: *p*-nitrophenyl.
(DOC)

## References

[1] Bowers WS, Nault LR, Webb RE, Dutky SR (1972). Aphid alarm pheromone: isolation, identification, synthesis.. Science.

[2] Edwards LJ, Siddall JB, Dunham LL, Uden P, Kislow CJ (1973). Trans-beta-farnesene, alarm pheromone of green peach aphid, *Myzus persicae* (Sulzer).. Nature.

[3] Francis F, Vandermoten S, Verheggen F, Lognay G, Haubruge E (2005). Is (*E*)-β-farnesene the only volatile terpenoid in aphids?. J Appl Entomol.

[4] Dewhirst SY, Pickett JA, Hardie J (2010). Aphid Pheromones.. Vitamins & Hormones.

[5] Dawson GW, Pickett JA, Smiley DW (1996). The aphid sex pheromone cyclopentanoids: synthesis in the elucidation of structure and biosynthetic pathways.. Bioorg Med Chem.

[6] Birkett MA, Pickett JA (2003). Aphid sex pheromones: from discovery to commercial production.. Phytochemistry.

[7] Griffiths DC, Pickett JA (1980). A potential application of aphid alarm pheromones.. Entomol Exp Appl.

[8] Gibson RW, Pickett JA (1983). Wild potato repels aphids by release of aphid alarm pheromone.. Nature.

[9] Beale MH, Birkett MA, Bruce TJ, Chamberlain K, Field LM (2006). Aphid alarm pheromone produced by transgenic plants affects aphid and parasitoid behavior.. Proc Natl Acad Sci USA.

[10] Vogt RG, Riddiford LM (1981). Pheromone binding and inactivation by moth antennae.. Nature.

[11] Vogt RG, Blomquist GJ, Vogt RG (2003). Biochemical Diversity of Odor Detection: OBPs, ODEs and SNMPs..

[12] Tegoni M, Campanacci V, Cambillau C (2004). Structural aspects of sexual attraction and chemical communication in insects.. Trends Biochem Sci.

[13] Pelosi P, Zhou JJ, Ban LP, Calvello M (2006). Soluble proteins in insect chemical communication.. Cell Mol Life Sci.

[14] Mohl C, Breer H, Krieger J (2002). Species-specific pheromonal compounds induce distinct conformational changes of pheromone binding protein subtypes from *Antheraea polyphemus*.. Invert Neurosci.

[15] Pophof B (2002). Moth pheromone binding proteins contribute to the excitation of olfactory receptor cells.. Naturwissenschaften.

[16] Pophof B (2004). Pheromone-binding proteins contribute to the activation of olfactory receptor neurons in the silkmoths *Antheraea polyphemus* and *Bombyx mori*.. Chem Senses.

[17] Grosse-Wilde E, Svatos A, Krieger J (2006). A pheromone binding protein mediates the bombykol-induced activation of a pheromone receptor in vitro.. Chem Senses.

[18] Forstner M, Breer H, Krieger J (2009). A receptor and binding protein interplay in the detection of a distinct pheromone component in the silkmoth *Antheraea polyphemus*.. Int J Biol Sci.

[19] Xu P, Atkinson R, Jones DN, Smith DP (2005). *Drosophila* OBP LUSH is required for activity of pheromone-sensitive neurons.. Neuron.

[20] Laughlin JD, Ha TS, Jones DNM, Smith DP (2008). Activation of pheromone-sensitive neurons is mediated by conformational activation of pheromone binding protein.. Cell.

[21] Matsuo T, Sugaya S, Yasukawa J, Aigaki T, Fuyama Y (2007). Odorant-binding proteins OBP57d and OBP57e affect taste perception and host-plant preference in *Drosophila sechellia*.. PLoS Biol.

[22] Swarup S, Williams TI, Anholt RR (2011). Functional dissection of Odorant binding protein genes in *Drosophila melanogaster*.. Genes Brain Behav.

[23] Biessmann H, Andronopoulou E, Biessmann MR, Douris V, Dimitratos SD (2010). The *Anopheles gambiae* Odorant Binding Protein 1 (AgamOBP1) Mediates Indole Recognition in the Antennae of Female Mosquitoes.. PLoS ONE.

[24] Pelletier J, Guidolin A, Syed Z, Cornel AJ, Leal WS (2010). Knockdown of a mosquito odorant-binding protein involved in the sensitive detection of oviposition attractants.. J Chem Ecol.

[25] Zhou JJ, Vieira FG, HeXL, Smadja C, Liu R (2010). Genome annotation and comparative analyses of the odorant-binding proteins and chemosensory proteins in the pea aphid *Acyrthosiphon pisum*.. Insect Mol Biol.

[26] Qiao HL, Tuccori E, He XL, Gazzano A, Field LM (2009). Discrimination of alarm pheromone (E)-β-farnesene by aphid odorant-binding proteins.. Insect Biochem Mol Biol.

[27] Sun Y, Qiao H, Ling Y, Yang S, Rui C (2011). New analogues of (E)-β-farnesene with insecticidal activity and binding affinity to aphid odorant-binding proteins.. J Agric Food Chem.

[28] Ban LP, Scaloni A, D'Ambrosio C, Zhang L, Yan YH (2003). Biochemical characterization and bacterial expression of an odorant-binding protein from *Locusta migratoria*.. Cell Mol Life Sci.

[29] Calvello M, Guerra N, Brandazza A, D'Ambrosio C, Scaloni A (2003). Soluble proteins of chemical communication in the social wasp *Polistes dominulus*.. Cell Mol Life Sci.

[30] Read DP, Feeny PP, Root RB (1970). Habitat selection by aphid parasite *Diaeretiella-rapae* (Hymenoptera-Braconidae) and hyperparasite *Charips-brassicae* (Hymenoptera-Cynipidae).. Can Entomol.

[31] Sakuma M, Fukami H (1985). The linear track olfactometer: an assay device for taxes of the German cockroach, *Blattella germanica* (L.) (Dictyoptera: Blattellidae) toward their aggregation pheromone.. Appl Entomol Zool.

[32] Siegel S, Castellan NJ (1988). Nonparametric statistics for the Behavioral Sciences.

[33] Schwede T, Kopp J, Guex N, Peitsch MC (2003). SWISS-MODEL: an automated protein homology-modeling server.. Nucleic Acids Res.

[34] Guex N, Peitsch MC (1997). SWISS-MODEL and the Swiss-PdbViewer: an environment for comparative protein modelling.. Electrophoresis.

[35] Arnold K, Bordoli L, Kopp J, Schwede T (2006). The SWISS-MODEL Workspace: a web-based environment for protein structure homology modelling.. Bioinformatics.

[36] Lartigue A, Gruez A, Spinelli S, Riviere S, Brossut R (2003). The crystal structure of a cockroach pheromone-binding protein suggests a new ligand binding and release mechanism.. J Biol Chem.

[37] Kruse SW, Zhao R, Smith DP, Jones DN (2003). Structure of a specific alcohol binding site defined by the odorant binding protein LUSH from *Drosophila melanogaster*.. Nat Struct Biol.

[38] Pickett JA, Griffiths DC (1980). Composition of aphid alarm pheromones.. J Chem Ecol.

[39] Hori M (2007). Onion aphid (*Neotoxoptera formosana*) attractants, in the headspace of *Allium fistulosum* and *A. tuberosum* leaves.. J Appl Entomol.

[40] Hori M (1998). Repellency of rosemary oil against *Myzus persicae* in a laboratory and in a screenhouse.. J Chem Ecol.

[41] Butt TM, Beckett A, Wilding N (1990). A histological study of the invasive and developmental processes of the aphid pathogen *Erynia neoaphidis* (Zygomycotina: Entomophthorales) in the pea aphid *Acyrthosiphon pisum*.. Can J Bot.

[42] Roy HE, Pell JK, Alderson PG (1999). Effects of Fungal Infection on the Alarm Response of Pea Aphids.. J Invert Pathol.

