# Supplementary figures and images for: Two Odorant-Binding Proteins Mediate the Behavioural Response of Aphids to the Alarm Pheromone (E)-ß-farnesene and Structural Analogues
Source: PLoS One. 2012 Mar 12;7(3):e32759. doi: 10.1371/journal.pone.0032759 (PMC3299684; doi:10.1371/journal.pone.0032759)

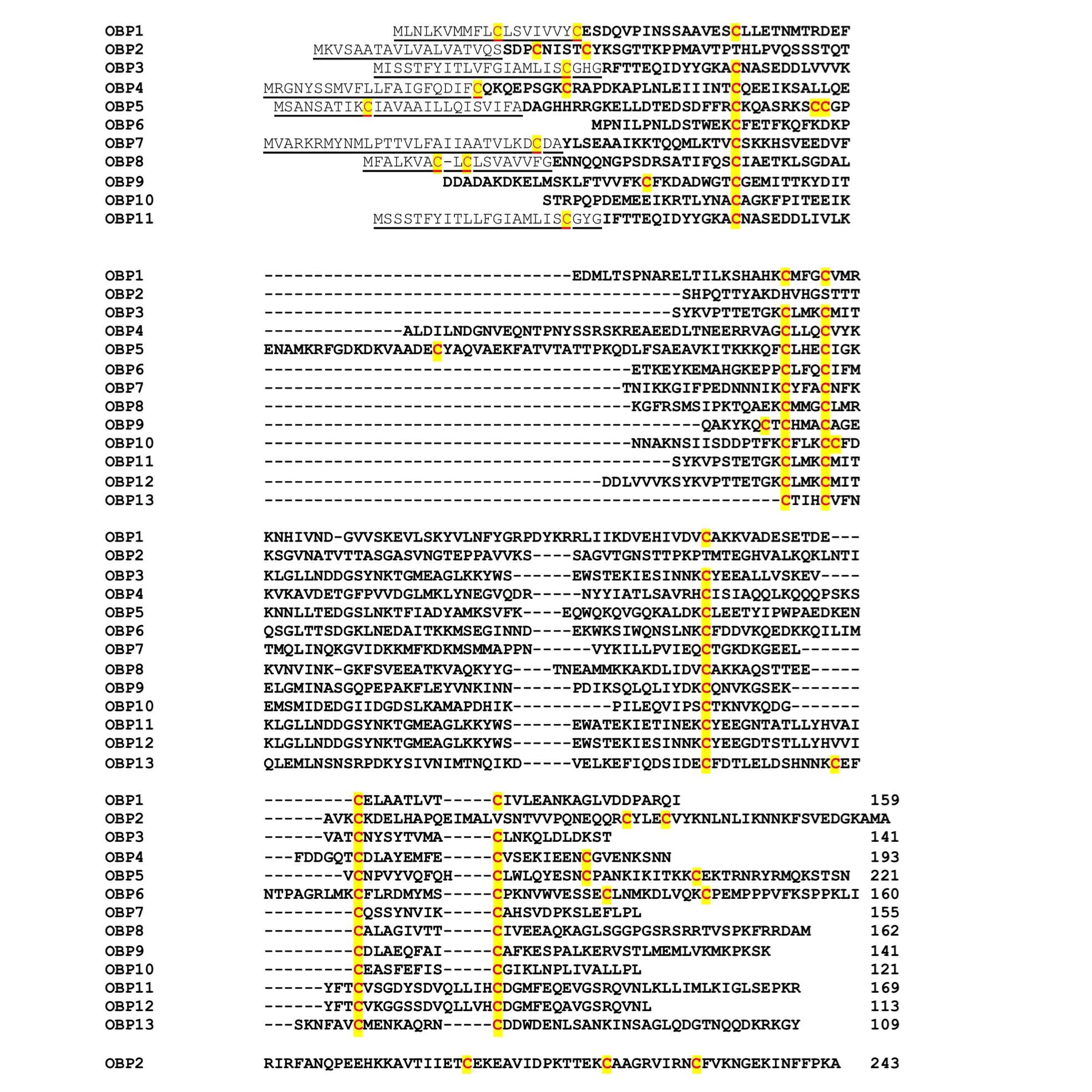

Supplement: Figure S1 — Alignment of amino acid sequences of the OBPs of A. pisum . The 13 sequences predicted by the genome are very divergent and, in order to align the six conserved cysteines, several gaps had to be introduced. OBP2 is much longer and, together with OBP4, OBP5 and OBP6, present several additional cysteines. Signal peptides (where prediction was feasible) are underlined. (TIFF) [file pone.0032759.s001.tiff]
