# Supplementary material for: Two Odorant-Binding Proteins Mediate the Behavioural Response of Aphids to the Alarm Pheromone (E)-ß-farnesene and Structural Analogues
Source: PLoS One. 2012 Mar 12;7(3):e32759. doi: 10.1371/journal.pone.0032759 (PMC3299684; doi:10.1371/journal.pone.0032759)
Supplement: Table S1 — Binding properties of the ligands utilised in ligand binding assays and behaviour experiments. For each protein, [IC]50 reports the concentration of ligand that halves the initial fluorescence intensity (both protein and 1-NPN were used at the concentration of 2 µM). Dissociation constants are calculated as reported in the Materials and Methods section. The structures of “CAU” ligands are reported in Table 1, those of “LIG” chemicals in Figure 3. Blank cells indicate values of [IC]50 higher than 30 µM. For these compounds dissociation constants have not been calculated. (DOC) [file pone.0032759.s002.doc]

**Table S1**. Binding properties of the ligands utilised in ligand binding assays and behaviour experiments. For each protein, [IC]_50_ reports the concentration of ligand that halves the initial fluorescence intensity (both protein and 1-NPN were used at the concentration of 2 μM). Dissociation constants are calculated as reported in the Materials and Methods section. The structures of “CAU” ligands are reported in Table 1, those of “LIG” chemicals in Figure 3. Blank cells indicate values of [IC]_50_ higher than 30 μM. For these compounds dissociation constants have not been calculated.

| Ligand | [IC]_50_ | | | | | | K_D_ | | | | | |
| --- | --- | --- | --- | --- | --- | --- | --- | --- | --- | --- | --- | --- |
|  | OBP1 | OBP3 | OBP6 | OBP7 | OBP8 | OBP10 | OBP1 | OBP3 | OBP6 | OBP7 | OBP8 | OBP10 |
| CAU-1 |  | 4.8 |  | 4.8 |  |  |  | 3.7 |  | 2.5 |  |  |
| CAU-2 | 10 | 3.9 | 12 | 2.8 | 8 | 20 | 8.1 | 3.0 | 10.3 | 1.4 | 6.0 | 8.8 |
| CAU-4 |  | 10 |  |  |  |  |  | 7.7 |  |  |  |  |
| CAU-7 |  | 12 |  |  |  |  |  | 9.2 |  |  |  |  |
| CAU-10 |  |  |  |  |  |  |  |  |  |  |  |  |
| CAU-14 | 5.8 | 3.2 | 6 | 2.8 | 12 | 20 | 4.7 | 2.4 | 5.2 | 1.4 | 9.0 | 8.8 |
| CAU-15 | 2.5 | 2.7 | 10.5 | 1.3 | 10.5 | 16 | 2.0 | 2.1 | 9.0 | 0.67 | 7.9 | 7.0 |
| CAU-16 |  | 4.6 |  | 8.2 | 13 |  |  | 3.5 |  | 4.2 | 9.8 |  |
| CAU-19 |  | 3.2 |  |  |  |  |  | 2.5 |  |  |  |  |
| CAU-21 |  | 8.1 |  | 11 |  |  |  | 6.2 |  | 5.7 |  |  |
| CAU-23 | 2.9 | 5.2 | 4.1 | 5 | 6.3 | 20 | 2.4 | 4.0 | 3.5 | 2.6 | 4.7 | 8.8 |
| CAU-24 |  |  |  |  |  |  |  |  |  |  |  |  |
| CAU-26 | 2.3 | 6.8 | 3.6 | 6.4 | 3.7 | 16.7 | 1.9 | 5.2 | 3.1 | 3.3 | 2.8 | 7.3 |
| CAU-27 |  | 16.5 |  | 1.1 |  |  |  | 12.6 |  | 0.57 |  |  |
| CAU-28 |  | 4.2 |  | 7.5 |  |  |  | 3.2 |  | 3.9 |  |  |
| CAU-35 |  | 4 |  |  |  |  |  | 3.1 |  |  |  |  |
| CAU-37 |  | 16 |  |  |  |  |  | 12.3 |  |  |  |  |
| CAU-42 |  | 3.6 |  | 6.6 |  |  |  | 2.8 |  | 3.4 |  |  |
| CAU-43 |  | 2.7 |  | 5.3 |  |  |  | 2.1 |  | 2.7 |  |  |
| CAU-46 |  | 1.9 |  |  |  |  |  | 1.5 |  |  |  |  |
| CAU-53 | 1.9 | 2.8 | 3.1 | 3.7 | 2.9 | 18 | 1.5 | 2.1 | 2.7 | 1.9 | 2.2 | 7.9 |
| LIG-1 |  | 17 |  | 2.2 |  |  |  | 13 |  | 1.3 |  |  |
| LIG-2 |  | 3 |  | 8.3 |  |  |  | 2.3 |  | 4.3 |  |  |
| LIG-3 |  | 18 |  | 16 |  |  |  | 13.8 |  | 8.2 |  |  |
| LIG-4 |  | 4.6 | 16.1 | 2.5 | 12.1 | 20 |  | 3.5 | 13.9 | 1.3 | 9.1 | 8.8 |
| LIG-5 |  | 8.9 | 26 | 8.4 | 17.2 | 25 |  | 6.8 | 22.4 | 4.3 | 13.0 | 11.0 |
| LIG-6 |  | 24 | 9.6 | 3.8 | 7.5 |  |  | 18.4 | 8.3 | 2.0 | 5.7 |  |
| LIG-7 |  |  |  | 18 |  |  |  |  |  | 9.3 |  |  |
| LIG-8 |  | 14 |  | 5.5 |  |  |  | 10.7 |  | 2.8 |  |  |
