# Supplementary material for: Two Odorant-Binding Proteins Mediate the Behavioural Response of Aphids to the Alarm Pheromone (E)-ß-farnesene and Structural Analogues
Source: PLoS One. 2012 Mar 12;7(3):e32759. doi: 10.1371/journal.pone.0032759 (PMC3299684; doi:10.1371/journal.pone.0032759)
Supplement: Table S2 — Comparison among behavioural responses elicited by all compounds tested separately or as a function of OBPs bound. Asterisks indicate that the difference is statistically significant: * P<0.05, ** P<0.01, ***P<0.001. (DOC) [file pone.0032759.s003.doc]

Table S2- Comparison among behavioural responses elicited by all compounds tested separately or as a function of OBPs bound. Asterisks indicate that the difference is statistically significant: * P<0.05, ** P<0.01, ***P<0.001.

| **aphid species** | **Comparison level** | **χ^2^** | **d.f.** | **significance** |
| --- | --- | --- | --- | --- |
| *M. persicae* | Among all ligands tested | 122.94 | 28 | *** |
|  | among all OBP3 and/or OBP7 ligands | 29.62 | 16 | * |
|  | among ligands grouped in function of affinity to OBP3, or OBP7, or both | 2.31 | 2 | N.S. |
|  | among ligands of additional OBPs | 4.26 | 11 | N.S |
|  | between OBP3 and/or OBP7 ligands as a whole and ligands of additional OBPs as a whole | 96.04 | 1 | *** |
| *A.pisum* | Among all ligands tested | 43.15 | 28 | * |
|  | among all OBP3 and/or OBP7 ligands | 10.93 | 16 | N.S |
|  | among ligands of additional OBPs | 14.30 | 11 | N.S |
|  | between OBP3 and/or OBP7 ligands as a whole and ligands of additional OBPs as a whole | 13.97 | 1 | *** |
