# Supplementary material for: Two Odorant-Binding Proteins Mediate the Behavioural Response of Aphids to the Alarm Pheromone (E)-ß-farnesene and Structural Analogues
Source: PLoS One. 2012 Mar 12;7(3):e32759. doi: 10.1371/journal.pone.0032759 (PMC3299684; doi:10.1371/journal.pone.0032759)
Supplement: Table S3 — Structures of the “CAU” ligand utilised in binding assays and behaviour experiments. Abbreviations are: Me: methyl; Pr: propyl; i-Pr: isopropyl; t-Bu: tert-butyl; Phe: phenyl; p-NO2: p-nitrophenyl. (DOC) [file pone.0032759.s004.doc]

**Table S3**. Structures of the “CAU” ligand utilised in binding assays and behaviour experiments. Abbreviations are: Me: methyl; Pr: propyl; i-Pr: isopropyl; t-Bu: *tert*-butyl; Phe: phenyl; p-NO_2_: *p*-nitrophenyl.

| Structure | Name | R_1_ | R_2_ | R_3_ |
| --- | --- | --- | --- | --- |
|  | CAU-1 | t-Bu | Me | H |
|  | CAU-2 | Phe | Me | H |
|  | CAU-4 | t-Bu | Pr | H |
|  | CAU-7 | t-Bu | i-Pr | H |
|  | CAU-10 | t-Bu | Phe | H |
|  | CAU-16 | t-Bu | Me | H |
|  | CAU-19 | t-Bu | Pr | H |
|  | CAU-21 | t-Bu | i-Pr | H |
|  | CAU-23 | Phe | i-Pr | H |
|  | CAU-24 | t-Bu | Phe | H |
|  | CAU-26 | pNO_2_ | Phe | H |
|  | CAU-35 | t-Bu | i-Pr | Cl |
|  | CAU-37 | t-Bu | Phe | Cl |
|  | CAU-42 | t-Bu | Pr | H |
|  | CAU-43 | t-Bu | i-Pr | H |
|  | CAU-46 | t-Bu | Phe | H |
|  | CAU-53 | pNO_2_ | Me | Cl |
|  | CAU-14 | R = *p*-bromophenyl | | |
|  | CAU-15 | R = 2,6-difluorophenyl | | |
|  |  |  |  |  |
|  |  |  |  |  |
|  | CAU-27 | R = *p*-*tert*-butylphenyl | | |
|  | CAU-28 | R = *p*-bromophenyl | | |
|  |  |  |  |  |
